# The use of dual mobility acetabular cups in total hip replacement reduces dislocation rates in hip dysplasia patients

**DOI:** 10.1038/s41598-023-49703-z

**Published:** 2023-12-16

**Authors:** Jung Shin Kim, Nam Hoon Moon, Min Uk Do, Sung Won Jung, Kuen Tak Suh, Won Chul Shin

**Affiliations:** 1 Department of Orthopaedic Surgery, Pusan National University Yangsan Hospital, Pusan National University School of Medicine, Yangsan, Republic of Korea; 2 Department of Orthopaedic Surgery, Pusan National University Hospital, Busan, Republic of Korea; 3 Department of Orthopaedic Surgery, Sehung Hospital, Busan, Republic of Korea

**Keywords:** Diseases, Medical research

## Abstract

Total hip replacement arthroplasty (THA) in hip dysplasia patients has a higher dislocation rate than in patients with simple hip osteoarthritis due to anatomical deformation. Therefore, to reduce postoperative THA dislocation is the challenge for arthroplasty surgeons. From 2015 to 2020, 1525 patients underwent THA performed by two surgeons at a single institution. A total of 152 patients involving 172 THAs were included. The patients were classified into dual-mobility (DM) and fixed-bearing (FB) acetabular cup groups. The occurrence of postoperative dislocation and functional evaluation of the hip joint, was analyzed before and after surgery using the modified Harris hip score(mHHS). There was no difference in the preoperative demographics and radiographic parameters between the groups. The incidence of postoperative hip dislocation was significantly lower in the DM group (DM 0% vs. FB 9.0%) (*P* value = 0.003). The mHHS showed no difference before surgery and after surgery (DM 91.80 vs FB 92.03). Treating hip dysplasia patients with THA using a dual-mobility acetabular cup can reduce postoperative dislocations, and could be used for the better management of these patients.

## Introduction

Hip dysplasia and femoral acetabular impingement syndrome are the main causes of secondary hip osteoarthritis in adults^1,2^. According to studies hip dysplasia accounts for approximately 20–40% of hip osteoarthritis patients^3,4^. It is known to promote mechanical stimulation in the cartilage layer of the acetabulum and femoral head, leading to osteoarthritis^5^. Patients with hip osteoarthritis due to hip dysplasia are associated with anatomical deformation around the hip joint. Most acetabular deformations are hypoplastic anteriorly and superiorly, resulting in insufficient hip bone coverage of the acetabulum^6^. The femur also shows a narrow intramedullary canal, with hypoplastic changes. Owing to torsion, the femur has excessive anteversion, and the greater trochanter is located posteriorly^7^. The incidence of dislocation or intraprosthetic dissociation after THA is high in patients with hip dysplasia due to insufficient bone stock, deformed anterior and posterior spaces due to excessive anteversion of the proximal femur, and acetabular dysplasia^8–11^.

THA is regarded as the most successful surgical treatment performed in the twenty-first century. However, fatal complications, such as infection, non-infectious dissociation, implant instability, and dislocation can occur. In the United States, the most common causes of revision total hip arthroplasty were instability/dislocation (22.5%), mechanical loosening (19.7%), and infection (14.8%). Instability/dislocation was also the most common reason for acetabular revision (33.0%) and isolated head and liner exchange (32.8%). Mechanical loosening was the most common indication for all-component revision (22.8%) and for isolated femoral component revision (24.7%)^12^.

The concept of DM acetabular cup was proposed in 1974 by Gilles Bousquet and Andre Rambert in France. It was designed by combining the principles of Charnley's low-friction arthroplasty and McKee–Farrar's concept of hip stability, which maximizes hip stability by increasing the femoral head-neck ratio. A movable polyethylene liner is placed on the DM acetabular cup as an additional bearing between the artificial femoral head and the acetabular cup^13–15^. However, THA using a DM acetabular cup is associated with dissociation between the implants and accelerated wear of the polyethylene liner of the acetabular cup due to the inherent dual articular surface^16^. Recently, vitamin E-infused highly cross-linked polyethylene (VEPE) became available in 2010, it is expected to be a suitable material for abrasion among polyethylene liners applied to DM acetabular cups^17^. Development of the polyethylene liner and the use of THA using a DM acetabular cup is increasing.

**Figure 1:**
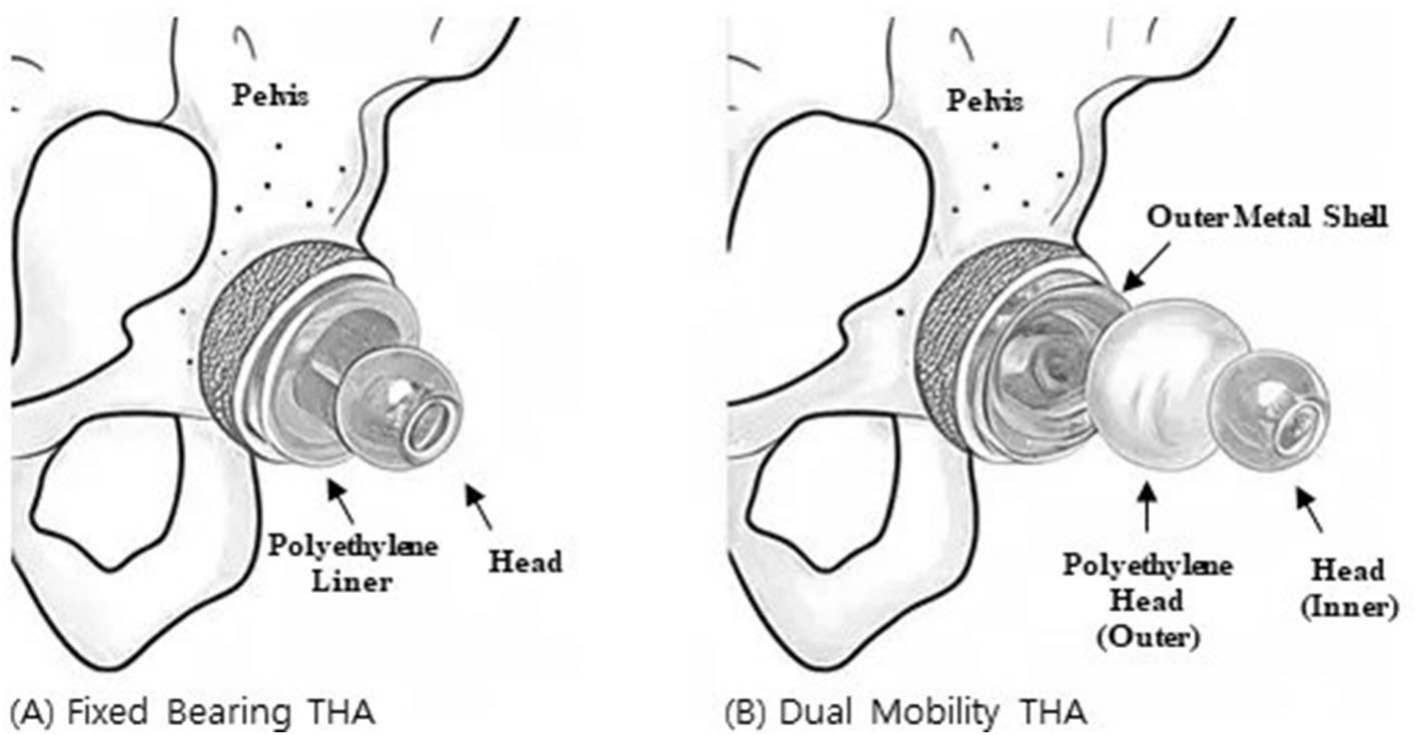
Schematic image of FB THA and DM THA. Schematic image of (**A**) FB THA, (**B**) DM THA drawn by J. S. Kim.

To date, few studies have directly compared THA using a DM acetabular cup and a FB acetabular cup in a high-risk patient group for dislocation (Fig. 1). Hence, we primarily aimed to evaluate the incidence of dislocation in patients with osteoarthritis due to hip dysplasia by comparing THA using a DM acetabular cup and FB acetabular cup. And secondary aimed to assess postoperative hip joint function. The authors hypothesized that postoperative dislocation was lower in the DM acetabular cup group and that there would be no significant difference in hip joint function between the two groups after surgery.

## Materials and methods

**Figure 2:**
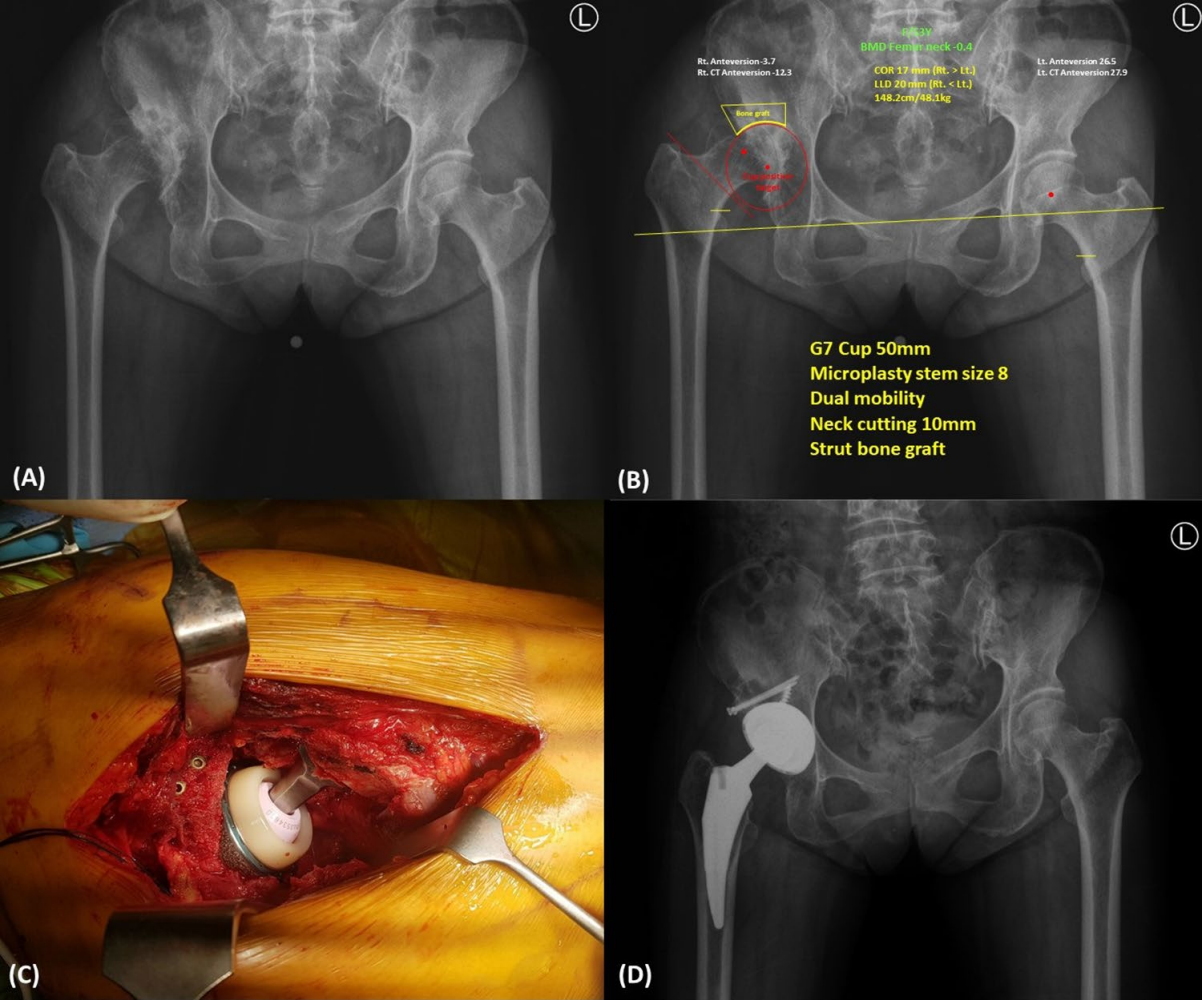
Sample images of DM THA in hip dysplasia patient. (**A**) Preoperative radiograph, (**B**) Templating using PACS, (**C**) Intraoperative photo, (**D**) Postoperative radiograph.

All surgeries were performed by two experienced arthroplasty surgeons at a single institution. All patients were treated under general anesthesia according to their condition, subject to anesthesiologic evaluation before surgery. Owing to the anatomical deformities caused by hip dysplasia, preoperative assessment and template using the picture archiving communication system (PACS) were performed. For the acetabular substitute, the position of the acetabular cup was targeted at an inclination angle of 40°and an anteversion angle of 20°, according to the Lewinnek safe zone (inclination 30°–50°, anteversion 5–25°)^18^. It was planned to sufficiently medialize the acetabular cup in the preoperative procedure so that more than 80% of the acetabular cup could be in contact with the osseous structure of the acetabulum (Fig. 2).

All patients were operated on in a lateral decubitus position using a posterolateral approach. Trilogy® (Zimmer/Biomet, Inc., Warsaw, IN) and G7® (Zimmer/Biomet) were used as FB acetabular cups and G7® was used as the DM acetabular cup. The acetabulum of a patient with hip dysplasia was shallow and hypoplastic in the anterior and superior directions; therefore, the acetabulum was prepared to be positioned medially, and the acetabular cup was press-fitted to the subchondral bone. For the initial mechanical stability of the acetabular cup, additional fixation was performed using one or two trans-acetabular screws.

When the acetabular cup did not come into contact with a sufficient area of the acetabular structure, it was fixed by reinforcing the osseous structure through a structural bone graft above the acetabulum. If acetabular cup coverage is insufficient by evaluating preoperative radiography and template, especially if there is no superior acetabular bone stock or in case of severe dysplasia, structural bone graft using femoral head is performed, and fixation with using cannulated screws. After bone graft fixation followed by acetabular reaming and cup position was executed. The femoral implant was selected according to the size of the intramedullary canal by deforming the patient's femur through a preoperative assessment Microplasty® (Zimmer/Biomet) and VerSys® (Zimmer/Biomet) were used as femoral implants, and Wagner cone® (Zimmer/Biomet) was used as a femoral implant in patients with severely narrow bone marrow. The length of the lower extremities was measured before surgery to minimize leg length discrepancies(LLD).

The maximum leg lengthening was set to 20 mm to minimize the occurrence of sciatic nerve paralysis. We evalutated preoperative templating and checked leg lengthening and femoral neck cutting level. During surgery, we measured leg lengthening with intraoperative ruler. After surgery we measured leg lengthening with preoperative radiography and postoperative radiography. During surgery, all hip joint stability tests (Hip flexion 90° & internal rotation 45°, hip flexion 40° & adduction & axial loading, hip extension & external rotation 40°) and Shuck test were performed after the artificial hip replacement was fixed. Finally, to prevent posterior dislocation, we performed short external rotator muscle and posterior hip joint capsule reconstruction using nonabsorbable sutures.

After the surgery, a hip abduction pillow was applied between patients’ lower extremities to maintain hip abduction. From the first day after surgery, lower-extremities quadriceps exercises and 3-point ambulation using crutches were performed. The range of motion was restricted until 3 weeks after surgery during which the patients did not perform hip flexion > 90°, adduction > 10°, and internal rotation > 10°. From 3 weeks after surgery, 90°of hip flexion was allowed, and ambulation using crutches was performed until 6 weeks. All the patients underwent the same postoperative rehabilitation program. After surgery, X-ray radiography was performed at an outpatient clinic every 3 weeks, 6 weeks, 3 months, 6 months, 12 months and annually.

**Figure 3:**
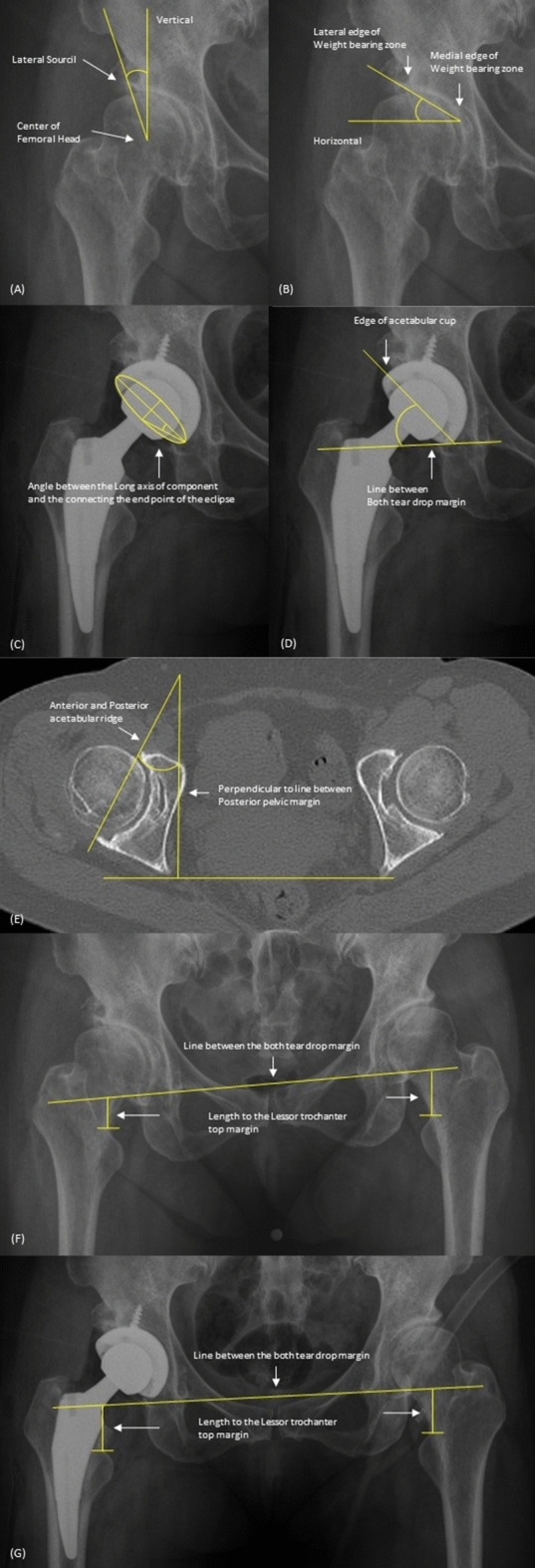
Radiographic measurement for preoperative evaluation and postoperative outcomes. (**A**) Mesurement of Lateral center edge angle (LCEA), (**B**) Mesurement of Tönnis angle, (**C**) Mesurement of angle for Liaw’s method, (**D**) Mesurement of cup inclination, (**E**) Mesurement of acetabular anteversion of CT, (**F**) Mesurement of preoperative Leg length discrepancy (LLD), (**G**) Mesurement of postoperative LLD.

To evaluate hip dysplasia, the lateral center edge angle (LCEA) and Tönnis angle were measured on preoperative true hip AP X-ray radiographs. Anteversion of the acetabulum was measured using a preoperative 3D hip computed tomography (CT) scan. The inclination of the acetabular cup was measured using a postoperative true hip AP radiography. After surgery, anteversion of the acetabular cup was measured using Liaw’s method, which is considered a relatively accurate method^19^. Indirect measurement of LLD was performed before and after surgery using true hip AP X-ray images and comparing the distance between the pelvic tear drop and the left and right lesser trochanters (Fig. 3).

The patients' surgical, anesthesia, medical, and imaging records were evaluated. Based on the implant used during THA surgery, the patients were classified into two groups: a group using a DM acetabular cup and a group using an FB acetabular cup. For postoperative comparison between the two groups, the incidence of dislocation was checked through medical and imaging records of the patients. To evaluate hip function after THA, the mHHS obtained before and until 2 years after surgery were analyzed.

### Statistical analyses

All data were expressed as the mean ± standard deviation. Kolmogorov–Smirnov and Shapiro–Wilk tests were used to examine the normal distribution of the data. The t-test was used to compare the means between the two groups. The chi-square test was used for frequency analysis of the data, and the Mann–Whitney test was used for scale analysis. Fisher's exact test was performed to conduct a comparative analysis of the dislocation rate between the two groups. For all statistical analyses, Statistical Product and Service Solutions software (SPSS) (version 26.0, IBM, Armonk, NY) was used, and a case with a *P* value < 0.05 was considered a statistically significant difference.

### Ethics approval and informed consent

This study followed the World Medical Association Declaration of Helsinki and strengthened the reporting of observational studies in epidemiology (STROBE) guidelines for cohort studies. All procedures performed in studies involving human participants were in accordance with ethical standards. The patient information was reviewed by the University Human Subjects Committee, and an informed consent exemption was obtained from the institutional review board (IRB) of our affiliated institutions (Pusan National University Yangsan Hospital, Approval No. 05-2022-173). All experimental protocols were approved by our institutional committee (Pusan National University Yangsan Hospital, Approval No. 05-2022-173).

## Results

**Figure 4:**
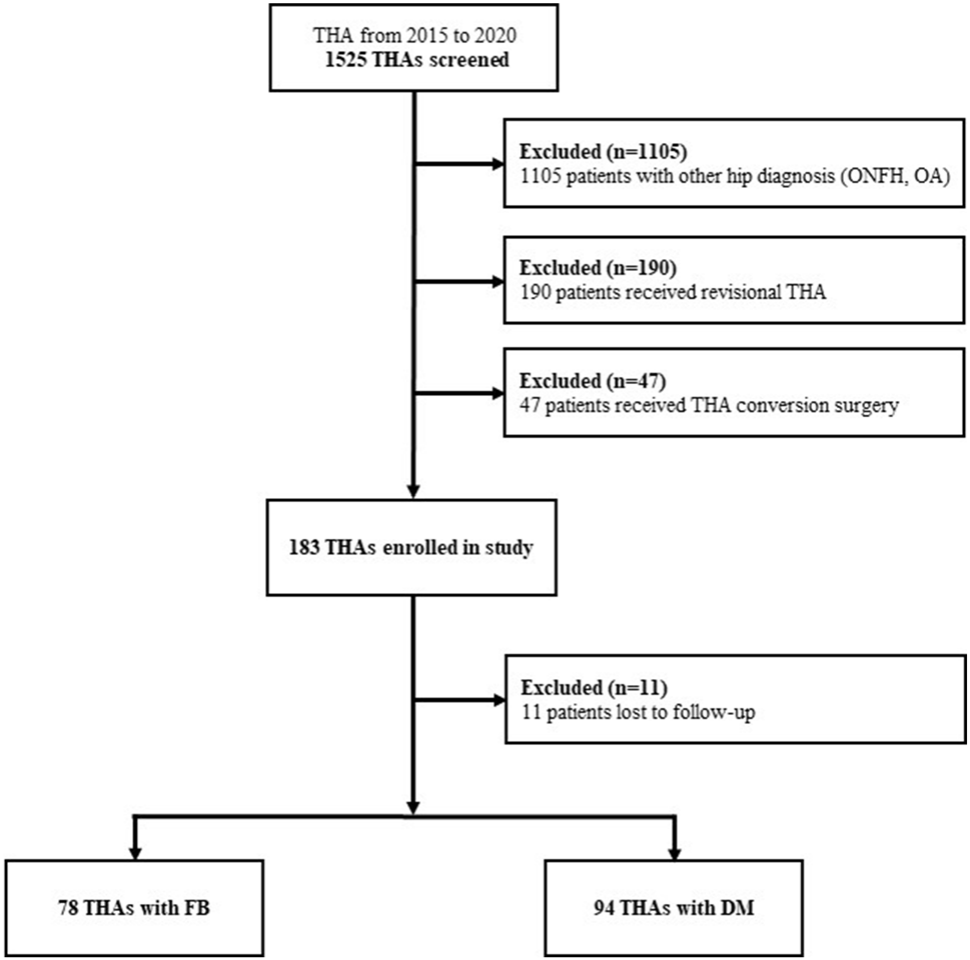
Flowchart of patient recruitment for this study.

This study included 1525 THA cases performed by two surgeons at a single institution between 2015 and 2020. Patients with inadequate follow-up, patients who underwent revision THA, prosthesis joint infection, or conversion to THA were excluded. Patients who underwent surgery for osteoarthritis due to hip dysplasia, and those who had a follow-up period of 2 years or more after surgery were enrolled. A total of 152 patients involving 172 THAs were included. Of the 172 THAs, 78 belonged to the FB acetabular cup group and 94 to the DM acetabular cup group (Fig. 4).

Among 172 cases, 46 were males and 126 were females. The mean age was 54.41 ± 12.29 years, the mean body mass index (BMI) was 24.36 ± 3.21, and the mean bone mineral density (BMD) was − 0.94 ± 1.06. There was no significant difference between the DM and FB groups (Table 1).

**Table 1:** Patient demographics.

	Overall	FB THA	DM THA	*P* values
All cases	172 (100)	78 (45.3)	94 (54.7)	
Sex				
Male	46 (26.7)	26 (15.1)	20 (11.6)	0.209*
Female	126 (73.3)	52 (30.2)	74 (43.0)	
Age (years)	54.41 ± 12.29	53.59 ± 12.57	56.70 ± 12.41	0.253^+^
Weight (kg)	62.06 ± 9.43	63.68 ± 9.33	60.05 ± 7.98	0.055^+^
Height (cm)	159.64 ± 8.61	160.79 ± 9.44	157.41 ± 7.21	0.063^+^
BMI (kg/m^2^)	24.36 ± 3.21	24.63 ± 2.84	24.32 ± 3.61	0.661^+^
BMD	− 0.94 ± 1.06	− 0.88 ± 1.14	− 1.24 ± 0.83	0.200^−^
Surgical side				
Right	94 (54.7)	34 (19.8)	60 (34.9)	0.094*
Left	78 (45.3)	44 (25.6)	34 (19.8)	

Values are presented as n (%) or mean ± SD. *P* values were derived from the *Chi-square test, ^+^independent t-test, and ^–^Mann–Whitney test to check whether the distribution was normal using the Kolmogorov–Smirnov and Shapiro–Wilk tests.

Regarding the Crowe classification based on the radiographic images of the hip joint, Crowe 1 was the most common in both groups, Crowe 2, Crowe 3, and Crowe 4 were distributed in the order (Table 2).

**Table 2:** Crowe classification of dysplastic hip between FB THA and DM THA groups.

	Overall	FB THA	DM THA
Crowe 1	91 (52.9)	40 (23.3)	51 (29.7)
Crowe 2	57 (33.1)	26 (15.1)	31 (18.0)
Crowe 3	19 (11.1)	9 (5.2)	10 (5.8)
Crowe 4	5 (2.9)	3 (1.7)	2 (1.2)

Values are presented as n (%).

All patients’ hip dysplasias were evaluated on preoperative radiological examination. The mean LCEA was 12.19° ± 4.25°, the average Tönnis angle was 27.71° ± 8.86°, and the mean anteversion angle on CT was 9.33° ± 12.51°. No statistically significant difference was observed between the DM and FB groups with respect to radiologic measurement (Table 3).

**Table 3:** Radiologic measurement of between FB THA and DM THA groups.

	Measured value			*P* value
	Overall	FB THA	DM THA	
Preoperative evaluations				
Lateral center edge angle (˚)	12.19 ± 4.25	12.01 ± 4.30	12.34 ± 4.24	0.724^+^
Tönnis angle (°)	27.71 ± 8.86	28.63 ± 8.81	26.96 ± 8.92	0.387^+^
CT anteversion (°)	9.33 ± 12.51	11.14 ± 8.89	7.82 ± 14.80	0.223^+^
Postoperative evaluations				
Liaw anteversion (°)	18.81 ± 4.48	19.47 ± 4.14	18.26 ± 4.71	0.214^+^
Cup inclination (°)	42.37 ± 4.62	42.96 ± 4.85	41.89 ± 4.41	0.285^+^
Leg length discrepancy				
Preoperative LLD (mm)	– 14.64 ± 13.43	– 10.56 ± 8.09	– 18.04 ± 15.91	0.009^+^
Postoperative LLD (mm)	2.57 ± 9.54	3.17 ± 5.36	2.07 ± 12.00	0.597^+^
LLD correction (mm)	17.48 ± 8.34	13.73 ± 6.27	20.58 ± 8.63	< 0.000^+^

Values are presented as n (%) or mean ± SD. *P* values were derived from the ^+^independent t-test. To check whether distribution was normal, we used the Kolmogorov–Smirnov and Shapiro–Wilk tests. *LLD* leg-length discrepancy.

After surgery, the average anteversion of the acetabular cup was 18.81° ± 4.48°, and the average acetabular cup inclination was 42.37° ± 4.62°. There was no statistically significant difference between the two groups in terms of these parameters. Before surgery, the LLD and an overall average of − 14.64 ± 13.43 mm for the affected lower extremity were observed. The average LLD was − 10.56 ± 8.09 mm and − 18.04 ± 15.91 mm in the FB and DM groups respectively, showing a significant difference between the two groups (*P*-value = 0.009). After surgery, the average LLD was observed (2.57 ± 9.54 mm), and there was no difference between the groups.

The size of the acetabular cup used during surgery and that of the femoral head implant were analyzed. The average sizes of the acetabular cup were 52.21 ± 3.34 mm and 51.95 ± 3.13 mm in the DM and FB groups respectively which were not statistically significant. Due to the nature of the DM acetabular cup, where the external polyethylene bearing acts as a substitute for the femoral head, and the size of the polyethylene bearing was compared with that of the replacement of the femoral head in the FB acetabular cup. The average sizes of the femoral head implant were 33.03 ± 1.99 mm, and 41.83 ± 2.66 mm in the FB and the DM acetabular cups respectively, showing a significant difference (*P*-value < 0.000) (Table 4).

**Table 4:** Comparison of Implant size and the number of Bone graft between FB THA and DM THA groups.

	Overall	FB THA	DM THA	*P* values
Cup (mm)	52.09 ± 0.35	51.95 ± 3.13	52.21 ± 3.34	0.708
Head (mm)	30.28 ± 0.31	33.03 ± 1.99	28.00	–
28 mm	94	–	94	–
32 mm	56	28	–	–
36 mm	22	11	–	–
DM bearing (mm)	–	–	41.83 ± 2.66	–
Head vs DM bearing	37.84 ± 0.54	33.03 ± 1.99	41.83 ± 2.66	< 0.000
Bone graft	10	6	4	–

Values are presented as n (%) or mean ± SD. *P* values were derived from independent t-test to check whether distribution was normal. We used the Kolmogorov–Smirnov and Shapiro–Wilk tests.

When analyzing the incidence of dislocations in both groups after THA, seven hip joints (9.0%) had dislocations only in the FB group. Among the patients who underwent surgery, 5 patients had 32 mm femoral head replacements and 2 patient had 36 mm femoral head replacements. In contrast, there was no dislocation in the DM group, and there was a statistically significant difference in the dislocation rate between the two groups (P-value = 0.003) (Table 5).

**Table 5:** Dislocation of FB THA versus DM THA in dysplastic hip patients.

	Overall	FB THA	DM THA	*P* value
No dislocation	165 (95.9)	71 (91.0)	94 (100.0)	0.003
Dislocation	7 (4.1)	7 (9.0)	0 (0)	

Values are presented as n (%), *P* value were derived from Fisher’s exact test.

**Figure 5:**
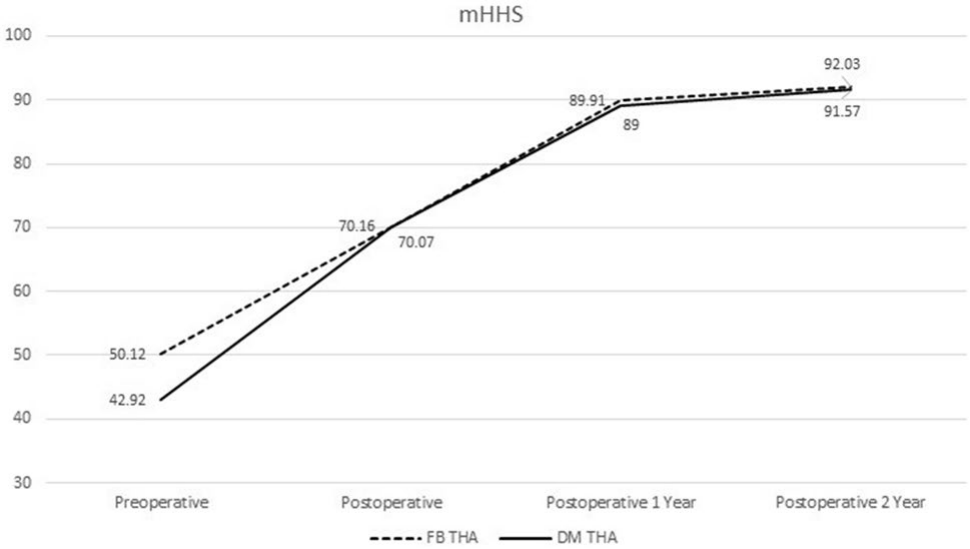
Graph of modified Harris hip score between the FB THA and DM THA groups.

In the preoperative evaluation of hip joint function using the mHHS, the FB group and the DM group showed average scores of 50.12 ± 17.82, and 42.92 ± 16.62 respectively, but there was no significant difference. The mHHS performed early postoperative, postoperative 1 year and 2 years after surgery showed no difference between the two groups (Table 6; Fig. 5).

**Table 6:** Comparison of modified harris hip score between FB THA and DM THA groups.

	Overall	FB THA	DM THA	*P* value
Preoperative	45.20 (17.35)	50.12 (17.82)	42.92 (16.66)	0.106
Postoperative	70.30 (7.72)	70.16 (8.59)	70.07 (7.08)	0.964
Postoperative 1 year	89.66 (4.35)	89.91 (4.93)	89.00 (3.88)	0.358
Postoperative 2 year	92.06 (4.91)	92.03 (5.41)	91.80 (4.25)	0.673

Values are presented as scores (SD). *P* values were derived from independent t-test. To check whether distribution was normal, we used the Kolmogorov–Smirnov and Shapiro–Wilk tests.

## Discussion

Hip dislocation after THA is one of the most common complications after surgery resulting in revisional THA^12,17^. Neuromuscular disorders, muscle weakness, hip dysplasia, abnormal spino-pelvic movement, previous hip fractures, and osteonecrosis of the femoral head are known as causative factors associated with a high risk of dislocation after surgery^20,21^. When performing THA in these patients, it is necessary to prepare for dislocation prevention.

Patients with osteoarthritis due to hip dysplasia have anatomical deformities around the hip joint which complicates THA. Most patients with hip dysplasia have hypoplasia of the acetabulum in the anterior and superior directions, resulting in insufficient hip-bone coverage of the acetabulum^6^. Due to hypoplasia, the acetabular cup used in THA must be small. Considering the period of use of the implant, to secure a sufficient thickness of the acetabular liner, there is no choice but to use only a smaller size. Consequently, the risk of hip dislocation increases with a decrease in the head-neck ratio and jump distance. The femur of patients with hip dysplasia has a narrow intramedullary canal and is hypoplastic^7^. The incidence of dislocation and dissociation of the prosthesis after THA is high in patients with hip dysplasia due to insufficient bone stock and deformed anterior and posterior spaces, excessive anteversion of the proximal femur, and dysplasia of the acetabulum^8–11^.

The artificial hip joint using the DM acetabular cup has two articular surfaces. Firstly, the articular surface between the small internal femoral head and the large hemispherical polyethylene implant, and secondly, the articular surface between the polyethylene insert and the external acetabular cup. The inner articular surface contributes to the major movement of the artificial joint, whereas the outer joint exhibits joint movement during an extreme range of movement^22^. The DM acetabular cup has a large femoral head replacement effect through a polyethylene bearing implant and has a large jump distance due to the increased effect of the head-neck ratio. This increases the stability of the artificial hip joint and the range of motion of the hip joint after surgery^23^.

Several studies have shown that the DM acetabular cup is associated with a decrease in the rate of dislocation and revisional surgery. According to Neri et al.^24^, in an original designer retrospective study on primary DM THA for 212 cases, showed excellent implant stability and survivorship with no dislocation at a mean follow-up of more than twenty-five years. Reina et al.^25^, compared 1583 THA using a DM group and FB group in 12 studies conducted from 2015 to 2018, wherein the dislocation rate of the DM and FB groups was 1%, and 7% respectively. Tarasevicius et al.^26,27^, evaluate DM THA dislocation rate in femoral neck fracture patient in 2010 and 2013. In 2010, FB and DM dislocation rate was 14.29% vs 0% (*P*-value = 0.01). And in 2013, a statistically significant decrease in the dislocation rate (10.4% vs. 0%, *P*-value = 0.01) was observed 1 year after surgery in the DM group of the study compared to the FB group with femoral neck fracture patients. Rowan et al.^28^ cunducted DM vs FB with THA in patients under 55 years, matched cohort analysis in 2017. In the DM group there were no dislocations and no intraprosthesis dislocation (IPD) at 3 years mean follow up. However, there were seven patients (5.1%) in the FB group that dislocated (*P*-value = 0.01). Zagorov et al.^29^ in 2018 compared postoperative dislocation rate with DM group with FB group and bipolar hemiarthroplasty for displaced femoral neck fratures. And concluded that DM group had 0% dislocation rate as compared to 11.1% in FB group and 3.1% in bipolar hemiarthroplasty. However, Agarwala et al.^30^ in 2021, a prospective cohort study of 103 eldery patients, compared DM group with FB group in femoral neck fracture patients with minimum follow up of 1 year. 52 patients treated with DM and 51 patients treated with FB. There was no dislocation occurred in both group, but range of motion was significantly better in DM group. There was no There was no difference in postoperative dislocation rate, postoperative stability, implant loosening, and rate of revision surgery between the two groups. But this study excluded of dysplastic patients and patients with neuromuscular disorder. Also Ciriello et al.^31^ in 2023, a retrospective comparative multicenter study, compared the dislocation rate of 129 THA using FB group with 133 THA using DM group. Dislocation rate of the FB and DM groups was 3.1%, and 1.5% respectively (*P*-value = 0.4416). the results of the study suggest that the use of DM in primary THA might ensure a lower risk of dislocation in comparison to FB group, although no statistical significant differences. And all the dislocation cases of DM group associated with IPD. Ciriello et al.^31^ concluded that, using DM in primary THA with caution only in high risk patients for dislocation, keeping in mind that dislocation and IPD can still occur with this type of implant, leading to survival rates for dislocation comparable to FB group (Table 7).

**Table 7:** The comparative studies evaluating the difference in dislocation rates and functional outcome between the FB THA and DM THA.

Study	Year	Sample size	Mean follow-up	Mean age in years	FB dislocation rate (%)	DM dislocation rate (%)	Difference in dislocation rate (P-value)	Difference in fuctional outcome (P-value)
Tarasevicious et al	2010	98	1 year	70.8	14.29	0	0.01	> 0.01
Tarasevicious et al	2013	125	1 year	75	10.4	0	0.01	> 0.01
Rowan et al	2017	272	3 years	48.5	5.1	0	0.01	> 0.01
Zogorov et al	2018	116	32 months	73.4	11.1	0	0.05	NA
Agarwala et al	2019	103	1 year	76.25	0	0	> 0.01	0.001
Ciriello et al	2023	262	29 months	71.4	3.1	1.5	0.44	NA
Present study	2023	172	2 years	54.41	9	0	< 0.01	> 0.01

In our study, the dislocation rate in FB group was 9% and 0% in DM group. Which is similar with Rowan et al. and Zogorov et al.’s previous study. all dislocations occurred in the FB group. In patients with dislocation, the size of the femoral head implant was 32 mm in five patients and 36 mm in two patients. Three patients were dislocated due to trauma within postoperative 6 months, and four patients experienced dislocations when they assumed an excessive hip flexion position after 1 year of surgery. Although it has been reported that the use of a femoral head implant of 32 mm or larger can reduce the occurrence of dislocation^17^, dislocation occurred even with the larger implant in patients at a high risk of dislocation. However, THA using a DM acetabular cup can prevent dislocation regardless of the size of the femoral head implant in patients with a high risk of dislocation^17^. The results of this study confirmed that the incidence of dislocation was significantly reduced in the DM group of patients with osteoarthritis due to hip dysplasia, as hypothesized by the authors.

IPD may occur as an inherent complication of DM acetabular cups. If the separation between the implants occurs in the DM acetabular cup, wear, metallization and collision of the femoral head may occur^32^. In our study, all 94 patients in the DM group in this study used third-generation highly cross-linked polyethylene inserts, and there was no IPD during the 2 years follow-up.

This study had several limitations. Firstly, since this was a single-institution, retrospective study, there is a possibility of selection bias in patient recruitment. Secondly, since this study analyzed the postoperative results of patients involving two surgeons, the generalizability of the study conclusions may be limited. Third, we couldn’t evaluate the difference and risk factors in each dislocated THA groups. Dislocation was occurred in FB group, 7 cases only. Lastly, in the evaluation of patients in this study, the follow-up period was short; therefore, long-term results could not be analyzed. Hence, studying the outcome of THA in patients with advanced hip dysplasia or the long-term outcomes after DM THA in these patients is necessary.

In this study, we found that postoperative dislocation can be reduced when THA using a DM acetabular cup is performed in patients with hip dysplasia who have a high risk of dislocation due to anatomical deformities. In addition, there was no difference in the functional outcome after THA between the DM and the FB group. Continuous follow-up of these patients is required to ensure objectivity in our results. However, we recommend using a DM acetabular cup for THA in patients with hip dysplasia.

## Data Availability

The data utilized are accessible from the corresponding author upon reasonable request.
